# Prevalence of Asymptomatic Group A *Streptococcus* Carriage Based on Rapid Antigen Detection Test in Healthy Adults in Poland

**DOI:** 10.3390/jcm14062008

**Published:** 2025-03-16

**Authors:** Martyna Biała, Patrycja Leśnik, Mateusz Babicki, Brygida Knysz

**Affiliations:** 1 Department of Infectious Diseases, Liver Diseases and Acquired Immune Deficiences, Wroclaw Medical University, 51-149 Wroclaw, Poland; 2 Department of Microbiology, Wroclaw Medical University, 50-368 Wroclaw, Poland; 3 Department of Family Medicine, Faculty of Medicine, Wroclaw Medical University, 51-141 Wroclaw, Poland

**Keywords:** *Streptococcus pyogenes*, GAS, carriage

## Abstract

**Background:** Acute pharyngitis is one of the most prevalent disorders seen in general practitioners’ consultations. Most cases of acute pharyngitis in adults are caused by respiratory viruses and are self-limited. However, clinical manifestations of viral pharyngitis can overlap with bacterial pharyngitis, mainly caused by group A *Streptococcus* (GAS). A rapid antigen test for GAS can help diagnose streptococcal pharyngitis, but misdiagnosing *S. pyogenes* infection in a patient with a viral condition can lead to inappropriate antibiotic use. Some patients with a sore throat due to a virus or other causes will test positive for GAS because of carriage. The aim of our study was to analyze rapid strep test results in healthy adults. **Methods:** A cohort study was conducted in an outpatient clinic in Wroclaw. We used the rapid strep test *BIOSYNEX STREP A*. **Results:** A total of 350 healthy volunteers (≥18 years old) were enrolled in this study. The presence of *Streptococcus pyogenes*, based on a rapid strep test, was detected in 17 adults (4.9%). The strep test positivity rate was higher in younger adults. **Conclusions:** In healthy individuals in Poland, the prevalence of pharyngeal carriage of GAS is 4.9%. This finding emphasizes that the rapid antigen detection test should only be used in cases of suspected bacterial pharyngitis to avoid inappropriate antibiotic use.

## 1. Introduction

Acute pharyngitis is one of the most prevalent disorders seen in general practitioners’ consultations. Among adults, the majority of cases of sore throat occur by age 40, and then incidence decreases with age [1]. Most cases of acute pharyngitis in adults are caused by respiratory viruses and are self-limited [2]. However, group A *Streptococcus* (GAS) is the predominant bacterial cause of acute pharyngitis and results in approximately 5–15% of cases of acute pharyngitis in adults in developed countries [2]. Patients with GAS usually demonstrate a sudden onset of sore throat, pharyngeal redness and swelling, patches or pus on tonsils, anterior cervical lymphadenopathy, fever, malaise and no cough. Clinical guidelines for pharyngitis recommend the administration of narrow-spectrum antibiotics when there is a strong, objective suspicion of GAS infection [3,4,5]. Despite this, antibiotics are frequently prescribed for patients with pharyngitis, even though most cases of sore throat are caused by seasonal viruses. The concern is that inappropriate antibiotic use may contribute to antimicrobial resistance, causing difficult-to-treat infections. It is challenging for clinicians to distinguish between viral and bacterial pharyngitis based on the clinical picture alone. Traditionally, culture-based methods need up to 48 h to grow. This has led to the evolution of rapid tests to detect GAS. The use of the Centor score and rapid strep test can enhance appropriate prescribing of antibiotics. However, we found that despite negative results of rapid strep tests, patients still received antibiotics [6]. Another study showed that 23% (80/345) of participants had a positive throat swab for GAS, but 65% (225/345) of all patients were prescribed immediate and 12% (43/345) delayed antibiotic regimens [7]. The authors of the indicated study also showed results from a retrospective report of the Centor score for 337 patients, 171 of whom had a Centor score between 0 and 2, and 166 had a Centor score of 3 or 4 [7]. Moreover, younger age is associated with a higher carriage rate, and the standard method used cannot distinguish between carriage and infection [7]. In Europe, a sharp increase in GAS infections—both invasive and non-invasive—has been observed since the autumn of 2022 in comparison to the COVID-19 pandemic and pre-pandemic periods [8,9,10]. The impact of this significant increase in GAS infections on asymptomatic *Streptococcus pyogenes* carriage in the general population is unknown. An asymptomatic person is considered to be a streptococcal pharyngeal carrier if they have a GAS-positive throat culture or rapid antigen detection test result without any symptoms of infection [11]. People who are GAS carriers have a lower pharyngeal density of *S. pyogenes* as opposed to those with an active infection due to GAS; this leads to a decreased potential for pathogen transmission [11]. The prevalence of GAS carriage in the pharynx also depends on factors including age, population and season [12]. Asymptomatic *S. pyogenes* carriage is the most widespread among children and adolescents [11]. However, little up-to-date data on post-COVID-19 pandemic changes in GAS carriage in children and adults is available. Misdiagnosing *S. pyogenes* carriage in patients with a viral condition could lead to the unnecessary prescribing of antibiotics to people who are likely suffering from viral pharyngitis with coincidental GAS carriage [13]. Confirmation of a true *S. pyogenes* infection with a typical clinical course requires both throat swab culture and a serological survey to identify participants with elevated antibodies targeting conserved GAS antigens, streptolysin-O and deoxyribonuclease-B [14,15]. However, serological surveys are not routinely performed in primary care, and we should also remember the disadvantages of serological methods, like cross-reactions, low performance and the window period. Moreover, in most of primary care clinics in Poland, throat cultures are also not routinely performed, but physicians do a rapid strep test to aid their decision-making. This study aims to analyze the rapid strep test results in healthy adults. We provide a hypothesis that the post-pandemic significant increase in GAS infections could change the rates of carriage in the population, and this can lead to a higher number of misdiagnosing *S. pyogenes* infection in patients with viral conditions and inappropriate antibiotic use in adults. In our analysis, we focused only on the rapid strep test routinely used by doctors who decide about antimicrobial treatment for pharyngitis in primary care settings.

## 2. Materials and Methods

A cohort study was conducted from April to June of the year 2024 in an outpatient clinic in Wroclaw. Of 380 people, 350 healthy volunteers (with no signs of pharyngitis) were enrolled in our study. We recruited adults during routine visits or people interested in participating in the study, including medical staff, their friends and families and students (Table 1).

Inclusion criteria were age ≥ 18 years old and the capability of giving signed informed consent. Adults were excluded from the study if they had signs or symptoms suggesting upper respiratory tract infections, had used antibiotics within 4 weeks before swabbing, did not consent to the study or were younger than 18 years. We used the rapid antigen detection test *BIOSYNEX STREP A* (Switzerland, France), which has a relative sensitivity of 94.4% (95%CI: 88.7–97.7%), relative specificity of 97.3% (95%CI: 95.1–98.6%) and accuracy of 96.6% (95%CI; 94.6–98.0%) [16]. The process of the rapid strep test was explained to each patient before throat swabbing. Throat swabs were collected by two physicians. We performed tests and interpreted the results according to the manufacturers’ instructions [16]. Adults were also asked about age, sex and COVID-19 and influenza vaccination status. Before sample collection, patients provided written informed consent. The study was approved by the Wroclaw Medical University Ethics Committee (KB-565/2023N). We divided the patients into two groups based on the results of the rapid strep test. For both groups—those with positive or negative rapid strep test results—we computed basic concepts like the number of cases, mean, median, range, lower and upper quartile and standard deviation of the ages. For the description of non-normally distributed data, the median (interquartile range [IQR]) was used. Absolute numbers and percentages were used for the description of qualitative variables. For qualitative parameters, the frequency of traits or characteristics in groups was analyzed using the χ^2^df test with the appropriate number of degrees of freedom, df (df = (m − 1) × (n − 1), where mis the number of rows and n is the number of columns). For non-parametric comparisons, we used the Mann-Whitney U test. All statistical calculations were completed using EPIINFO Ver. 7.2.3.1 and Statistica Ver. 13.3. *p*-values < 0.05 were considered significant.

## 3. Results

Out of 350 healthy volunteers, 133 (38%) were female and 217 (62%) were male. The age of the study group ranged from 18 to 64 years, with a median age of 31 years (IQR: 22–39). Only 56 (16%) patients declared that they had received a protective vaccination against the influenza virus prior the flu season. However, 342 (97.7%) reported having received at least three doses of a COVID-19 vaccine. The presence of *Streptococcus pyogenes*, based on the results of the rapid strep test (manufacture: *BIOSYNEX STREP A*), was detected in 17 adults (4.9%). Statistical analysis indicated no significant difference between positive rapid strep test results and patients’ sex (*p* = 0.782) or vaccination status (influenza *p* = 0.243, COVID-19 *p* = 0.518) (Table 2).

The strep test positivity rate was higher in younger adults (*p* = 0.0494), with a median age of 24 (IQR: 22–30) in the positive strep test group and 32 years (IQR: 22–39) in the negative strep test group.

## 4. Discussion

Detection of GAS in the pharynx verifies its presence, but not its role—whether it is carriage or active infection [17]. Without a clinical correlation of the findings, a positive rapid strep test result is not diagnostic of GAS pharyngitis. Moreover, Cohen JF et al. assessed the factors that influence the sensitivity of a rapid strep antigen test in children, including clinical manifestation, bacterial inoculum size and physician-related factors [17]. They showed that the sensitivity of a rapid strep test ranged from 56 to 96% among medical practitioners, likely due to a suboptimal throat swab technique and it was higher among physicians with hospital work experience compared to those in outpatient practice [17]. The rapid antigen strep test sensitivity was higher in children with pharyngitis than in asymptomatic children (89% vs. 41%) and in those with heavy bacterial inoculum (94% vs. 53%) [17]. Physicians should follow up a negative rapid test result in symptomatic children with a throat culture [4]. Administering antibiotics to children with confirmed streptococcal infection can reduce the risk of complications such as acute rheumatic fever [4]. However, throat culture is impractical for routine practice because it requires up to 48 h to grow. In adults, a throat culture after a negative rapid strep test is not routinely indicated because acute rheumatic fever is rare in this population [4]. The rate of GAS carriage in adults is lower than in children. However, basing diagnosis solely on a positive rapid strep test result may lead to unnecessary treatment of GAS carriers. Our study showed that *Streptococcus pyogenes* was detected in the pharynx of 4.9% of healthy volunteers based on rapid strep test results. Cunha BA described a case report of a 20-year-old college student suffering from a sore throat and mild malaise for three days [18]. The patient was apyretic, and his clinical findings were not specific for GAS-related infection—he had no fever, no pharyngeal edema, no patches or pus on tonsils and no swollen anterior cervical nodes [18]. Despite this, a rapid strep test was performed and the result was positive [18]. The patient was treated with antibiotics, but was later admitted to the hospital with severe herpes gingivostomatitis three days later [18]. In this case, the doctor did not consider GAS colonization in the pharynx, even though there were no clinical symptoms suggesting GAS pharyngitis. Respiratory viruses are the predominant cause of acute pharyngitis. Epstein-Barr virus and other herpes viruses (cytomaglovirus (CMV), herpes simplex virus) can also cause pharyngitis [2]. HIV and other sexually transmitted infections (gonorrhea, syphilis) are infrequent causes of pharyngitis; however, their rate increases markedly in high-risk groups, such as men who have sex with men (MSM) [2]. Guidelines recommend against streptococcal testing in patients with viral symptoms, but data showed that despite a low prevalence of GAS pharyngitis, testing for GAS was frequently performed in patients with viral conditions [19,20]. Differentiating viral from bacterial pharyngitis is challenging, but the Centor score (<2) or the NICE FeverPain score (<1) may be used to identify individuals who are unlikely to have group A streptococcal pharyngitis and therefore need not undergo a rapid strep test and are unlikely to benefit from antibiotic prescription [3]. Some patients with a sore throat due to viral infections or other causes (e.g., allergic reactions including rhinitis or sinusitis, cigarettes, gastroesophageal reflux disease [GERD], inhalation of dry air or trauma) will test positive for GAS because of carriage. Proper interpretation of rapid strep test results and clinical findings remains an important target for antimicrobial stewardship. The epidemiological context should always be considered when interpreting rapid antigen detection test results. The tests should be performed and interpreted according to the manufacturers’ instructions. Asymptomatic GAS carriage in the pharynx is uncommon in adults. However, a study conducted among Royal Marines recruits showed that despite a low frequency of GAS carriage, there is a probability of quick dissemination of this pathogen during outbreaks. At the beginning of the study, no carrier was detected in a small cohort (0/46), but at week 6, there were 8 cases of GAS carriage (17%) [21]. Additionally, Cordery R et al. indicated that despite treatment of cases and introduction of infection control methods like isolation and proper hygiene during the rise of scarlet fever in schoolchildren in the UK in 2018–2019, the rate of asymptomatic carriage of the outbreak strain increased among classroom contacts [22]. They observed a GAS carriage rate fluctuation of 10% in week 1, 27% in week 2, 24% in week 3 and 14% in week 4 [22]. Moreover, in two classrooms, bacterial settle plates were placed in elevated locations, and microbiological analysis showed that 17% and 50% of settle plates were positive for the outbreak strain, respectively [22]. We provide a hypothesis that the significant post-pandemic increase in GAS infections could change carriage rates in the general population; however, there is no up-to-date data in this field. Our small cohort suggests that several percent of healthy adults can be carriers. These results are similar to pre-pandemic data [13,23]. Furthermore, our study indicates a low influenza vaccination rate. Physicians should use motivational interviewing during patient consultations to encourage them for vaccinations.

Limitations: Our cohort is not representative of the entire adult population in Poland. Due to a relatively small sample size and single-site recruitment, results might differ in other regions of the country. We analyzed only the results of the rapid strep test; we did not perform a throat culture to compare the methods; however, we aimed to analyze routinely used methods in primary care in Poland. Moreover, we used a rapid strep test with a relative sensitivity of 94.4%, relative specificity of 97.3% and accuracy of 96.6%, so we cannot exclude false-positive or false-negative results. Another limitation is the absence of a group of patients diagnosed with pharyngitis; therefore, it is not possible to try to determine the clinical utility of this antigen test. We did not analyze the prevalence of chronic diseases in adults. We also did not analyze daily medication use or, if necessary, additional agents like topical use of antiseptics, e.g., octenidine.

## 5. Conclusions

The prevalence of pharyngeal carriage of GAS (based on the rapid antigen strep test) was 4.9% in healthy adults in Poland. Our results may be valuable for physicians and underscore the need for caution in interpreting rapid strep test results. This finding emphasizes that the rapid antigen detection test should only be used in cases of suspected bacterial pharyngitis to avoid inappropriate antibiotic use.

## Figures and Tables

**Table 1:** Groups of healthy adults included in the study.

Groups of Healthy Adults:	Total Number of Adults:	Excluded from the Study:	Included in the Study:
(1) Patients during routine visits	280	23	257
(2) Medical staff and their friends and families	63	5	58
(3) Students	37	2	35
Total:	380	30	350

**Table 2:** Analysis of rapid strep test results and patients’ sex and vaccination status.

Indicators:	Positive Rapid Strep Test Results (n)	Negative Rapid Strep Test Results (n)	*p* Value
Sex:			0.782
Women	7	126	
Men	10	207	
Vaccination status against influenza:			0.243
- Vaccinated	1	55	
- No Vaccination	16	278	
Vaccination status against COVID-19:			0.518
- Vaccinated	17	325	
- No Vaccination	0	8	

## Data Availability

The data presented in this study are available and can be shared on reasonable request sent to the corresponding author.
